# Acupuncture for mammary gland hyperplasia: A Bayesian network meta-analysis protocol

**DOI:** 10.1097/MD.0000000000022055

**Published:** 2020-09-04

**Authors:** Jun Xiong, Honglian Li, Fanghui Hua, Shouqiang Huang, Jie Xiang, Yunfeng Jiang, Kai Liao, Xiaohong Zhou, Meihua Liu

**Affiliations:** a The Affiliated Hospital of Jiangxi University of Traditional Chinese Medicine, Nanchang; b Haiyang People's Hospital of Shandong Province, Haiyang; c Jiangxi University of Traditional Chinese Medicine, Nanchang, China.

**Keywords:** acupuncture, mammary gland hyperplasia, meta-analysis, network, protocol

## Abstract

**Background:**

Multiple randomized controlled trials have shown that acupuncture (ACU) work well in the treating mammary gland hyperplasia, which has been widely used in hospitals of China. Although the choices of ACU treatments varies in practice, most are based on experience or preference. Therefore, we outline a plan to assess and rank the efficacy of the various ACU methods to formulate a prioritized regimen for mammary gland hyperplasia in ACU therapy.

**Methods:**

We will make a comprehensive retrieval in 7 databases as following: PubMed, Embase, Cochrane Library, China BioMedical Literature, China National Knowledge Infrastructure, Chinese Scientific Journals Database, and Wanfang database. The time is limited from the construction of the library to June 2020. We will evaluate the quality and the evidence of the included randomized controlled trials by the risk of bias tool and grading of recommendations assessment, development and evaluation, respectively. Bayesian network meta-analysis will be conducted using Stata16.0 and WinBUGS V.1.4.3.

**Results:**

The results of this study will be published in a peer-reviewed journal.

**Conclusions:**

Our study is expected to provide high-quality, evidence-based recommendations on further treatment of MGH for clinicians.

**Registration:**

PROSPERO (registration number CRD42020158743).

## 1 Introduction

Mammary gland hyperplasia (MGH) is a noninflammatory proliferative disease mostly occurring in women aged 25 to 45.^[1]^ Patients who have MGH can feel pain in their breasts as well as the breast lumps.^[2]^ MGH is a common disease among young and middle-aged women, accounting for more than 70% of all cases of breast disease, which seriously affects the normal life of patients.^[3]^

In recent years, more and more attention has been paid to the carcinogenesis tendency of MGH. The incidence of cancerization is 2% to 4%.^[4]^ In the United States, the rate of breast cancer in women whose biopsies showed proliferative disease with atypia is nearly 3-fold that of the women who had nonproliferative disease.^[5]^ Thus, the treatment of MGH is believed to be an effective approach for breast cancer prevention.^[6]^ Perhaps due to the fast pace of modern life, alongside increasing work-related stress and competitive pressure, the rate of occurrence of MGH has increased.^[7]^ The incidence of MGH in our country reached 25.5%, among which the number of people suffering from MGH accounted for the first place of mammary gland disease, reaching 78.6% of the number of people suffering from mammary gland disease, which was the high incidence among healthy people.^[8,9]^

Nowadays, it has become a generally accepted view that the occurrence of this disease is related to the endocrine disorder and mental factor. Periodic hormonal imbalance and(or) increased sensitivity of breast tissue to hormones are the leading causes of this disease.^[10]^ Modern medicine generally considers MGH to be related to sex hormone disorders secondary to dysfunction of the hypothalamus-pituitary-ovarian axis.^[11]^

Western medicine commonly used in the treatment of MGH include hormone preparations, hormone receptor inhibitors, Vitamins, and prolactin inhibitors.^[12]^ Hormone therapy or endocrinal therapy is often used to relieve symptoms over a short period. However, the side effects and complications of those treatments are severe, and the curative effect in long term application is also doubtful, such as delayed menstruation or menopause, increased leucorrhea, hot flashes, nausea, and so on.^[13]^ Vitamins are safe, easy to take and cheap, but the pain recurrence rate of it is high. Bromocriptine(a kind of prolactin inhibitors) is highly effective, but a long-term high dose of it can cause pulmonary, pleural, and retroperitoneal fibrosis.^[14]^ MGH itself has no indication of surgical treatment, and the operation often involves unnecessary injury or sacrifice of the breast, which is hardly accepted by the majority with a high recurrence rate. Thus, many patients need complementary and alternative therapy, such as acupuncture (ACU), which is generally considered to be safe and efficient by the populace.

MGH, also known as breast dysphasia, is classified under the “Rupi” category in Chinese medicine. Based on the traditional Chinese medicine theory, the pathogenesis is the disharmony of Chong and Ren, the stagnation of the liver, and the stasis of phlegm and blood. The treatment principles are to balance Chong and Ren, improve liver and kidney, and dredge phlegm and blood stasis.^[15]^ Modern research and clinical experiences of Traditional Chinese Medicine (TCM) doctors have also shown that the diseased kidney and conception and thoroughfare vessels are the root cause, that liver Qi stagnation, blood stasis, and phlegm are the symptomatic manifestations, and that they have a mutual cause-and-effect relationship.^[16]^

Nowadays, there is increasing scientific evidence suggests that ACU is beneficial for MGH. It has been found that ACU can regulate estrogen secretion level, make a benign effect on estrogen signal transduction pathway, inhibit estrogen promoting breast hyperplasia, thereby inhibiting the replication of breast proliferative cells, slowing down cell proliferation, and promoting cell apoptosis.^[17]^ Also, it can effectively speed up the blood flow in the lesion area, reduce the resistance index of blood flow, and improve the blood circulation of breast tissue.^[18]^

Presently, there was a network meta-analysis (NMA) of ACU for MGH, which is an English article.^[19]^ It compared the effectiveness of ACU and other therapies for MGH, not between various ACU treatments. Besides, it had deficiencies such as few outcome indicators and single search only in Chinese database. Hence, the aim of this study is to renew, improve, and strictly compare various ACU therapies to optimize ACU treatment of MGH and provide a foundational reference for clinical guide.

## 2 Methods

### 2.1 Study registration

This protocol report is structured according to the Preferred Reporting Items for Systematic Reviews and Meta-analysis Protocols (PRISMA-P) statement.^[20]^ It is registered on the International Prospective Register of Systematic Reviews (PROSPERO no. CRD42020158743; in PROSPERO 2020; http://www.crd.york.ac.uk/PROSPERO/display_record.php?ID=CRD42020158743.

### 2.2 Inclusion criteria

#### 2.2.1 Type of study

All the randomized controlled trials which is stated the “randomization” phrase will be included, regardless of allocation concealment, or used of blinding. The language will be restricted in Chinese or English.

#### 2.2.2 Types of participants

All patients, who are over 18, have MGH (as diagnosed using any authoritative diagnostic criteria) regardless of age, sex, race, duration of disease, weight, or education.

#### 2.2.3 Types of Interventions

ACU treatments include moxibustion, catgut embedding, electro-ACU, transcutaneous electrical acupoint stimulation, auricular ACU, scalp ACU, warm needling, manual ACU, acupoint injection, regardless of needling techniques and stimulation method.

#### 2.2.4 Types of control groups

The control group is treated with sham-ACU, placebo, pharmacotherapy, which is recommended in international or domestic authorized clinical guidelines, or no treatment. When studies combine ACU treatments with other active therapy, both the experimental and the control groups are required to use the same active therapy.

#### 2.2.5 Outcomes

##### 2.2.5.1 Primary outcomes

The effective rate.

##### 2.2.5.2 Secondary outcomes

Secondary outcomes will contain:

(1) Numerical rating scale: in the most painful day of breast pain before treatment and after treatment.
(2) Breast lumps size score: compare the size of the target mass before treatment and after treatment.
(3) Symptom disappearance rate (such as swelling pain, tenderness, emotional depression, hot temper, chest distress, irregular menstruation, and so on).
(4) Quality of life (QoL) measures of general health status, for example, the MOS 36-Item Short Form Health Survey (SF-36).
(5) Adverse effects (such as skin or tissue damage, pain or discomfort, nausea, dizziness, irregular menstruation).

### 2.3 Exclusion criteria

The exclusion certain contain the following items:

(1) Patients with acute medical conditions or pregnancy.
(2) Intervention combined with any complementary therapy will be excluded, for example, Chinese herb decoction, ACU, and other complementary therapy.
(3) Non-randomized controlled trials reviews, animal experiments, case report, expert experience, and conference article.
(4) Incomplete data or information.
(5) Repeatedly checked or published literature.

### 2.4 Search strategy

We will search the following electronic bibliographic databases: PubMed, Cochrane Library, EMBASE, Chinese National Knowledge Infrastructure, Chinese Biomedical Literature Database, Wanfang Database, the Chongqing VIP Chinese Science and Technology Periodical Database. All of them will be searched from inception to June 2020. The retrieval mode used will be a combination of free words and medical subject headings terms, including “fibrocystic breast disease,” “fibrocystic disease of breast,” “breast hyperplastic disease,” “hyperplasia of mammary glands,” “mammary glands hyperplasia,” “cyclomastopathy,” “acupuncture,” “acupuncture therapy,” “electroacupuncture,” “auriculotherapy,” “acupoint,” “acupoint injection,” “acupoint catgut embedding,” “moxibustion,” “needle,” “warm needle,” “temperature needle” “randomized controlled trial,” “randomized controlled,” “randomized, controlled,” “clinical trial.” The search strategy takes PubMed as an example, as shown in Table 1.

**Table 1:**
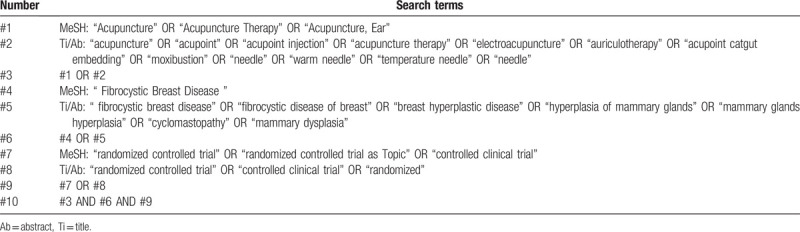
Search Strategy (PubMed).

### 2.5 Data collection and analysis

#### 2.5.1 Selection of studies

**Figure 1:**
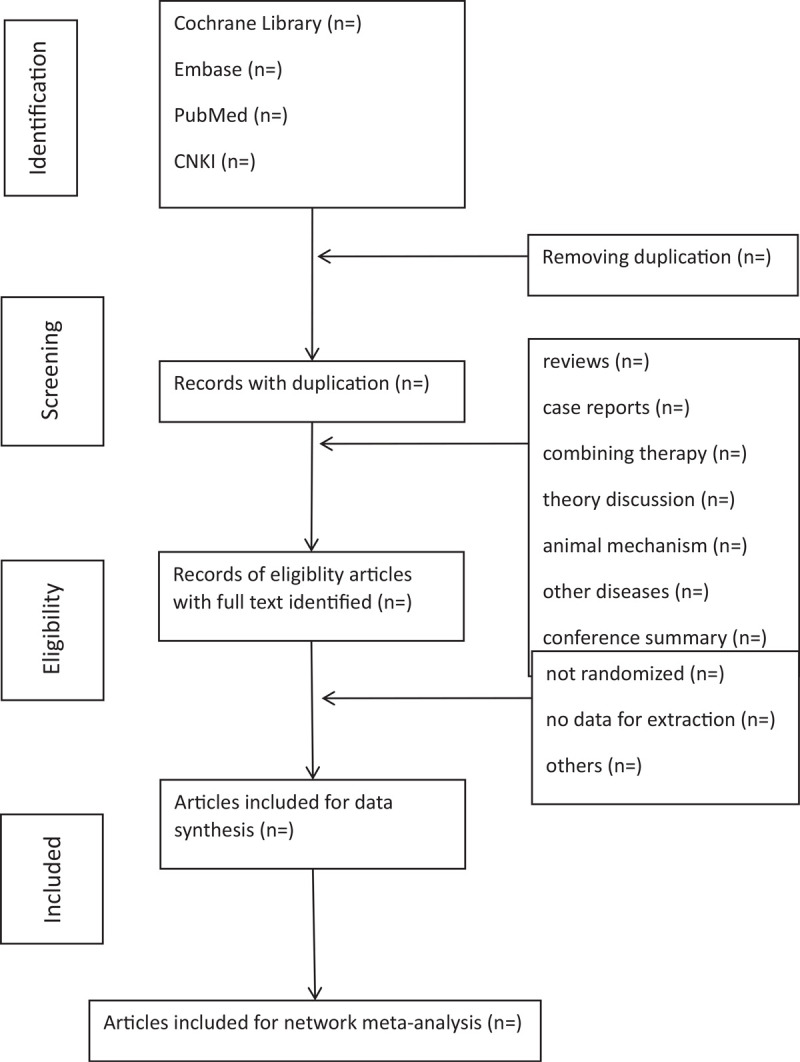
Flowchart of literature selection.

The selection, which includes literature screening, data extraction, and examination, will be conducted by 2 reviewers (QSH and JX). If there is any disagreement, a third reviewer (YFJ) will participate in the consultation to reach a consensus. The titles and abstracts of retrieved studies should be read first to rule out unrelated or repeated studies. Then, the remaining articles will be reviewed in full text by the reviewer according to the inclusion criteria, and the researches that meet the criteria will be selected at last. We will use the PRISMA flowchart to represent the complete process, which is shown in a PRISMA flow chart (Fig. 1).

#### 2.5.2 Data extraction and management

Two independent reviewers (QSH and HFH) shall design a standardized data extraction form, which including identification information (author, working location, publication date, publication source, etc), characteristic of trial (number of groups and participants, randomized method, blinding, etc), participants (age, sex, original disease, country, diagnosis, duration, etc), interventions of both the observation group and control group (type of ACU, frequency, session, duration), outcomes (primary outcome and secondary outcome, time of measurement, length of follow-up, etc), adverse effects, duration of follow-up, type and source of financial support. In case of any discrepancies between 2 reviewers, we will discuss and reach a consensus, and if necessary, seek arbitration from a third reviewer (JX).

#### 2.5.3 Dealing with missing data

If there are ambiguous or unreported data, we will contact the corresponding authors for the missing data to get specific information by telephone or email. If the missing information still cannot be obtained that missing information, we will exclude it from the analysis.

### 2.6 Risk of bias assessment

The assessment will be conducted by 2 reviewers (XHZ and KL) with the risk-of-bias assessment method from Cochrane Reviewer's Handbook 5.0.24.^[21]^ The main contents comprise 7 items: random sequence generation, allocation concealment, blinding of participants and personnel, blinding of outcome assessment, incomplete outcome data, selective reporting, and other sources of bias. The studies will be evaluated as being of “low risk of bias,” “high risk of bias,” or “unclear risk of bias.” Inconsistency will be consulted with the third review author (HLL).

### 2.7 Statistical analysis

#### 2.7.1 Pairwise meta-analysis

Continuous outcomes will be pooled with standardized mean differences with 95% confidence interval (95% CI), and dichotomous outcomes will be analyzed by calculating odds ratios with 95% CI. The *I*^2^ statistic will be used to assess levels of the heterogeneity of each pairwise comparison. If *I*^2^ < 50%, a fixed-effect model will be used, or else a random-effect model will be used. When the results are substantial heterogeneity or considerable heterogeneity, sensitivity analysis or meta-regression and subgroup analysis will be made to explore possible sources. When there is no explanation for statistical heterogeneity, a random-effects model will be used with a test level of *a* = 0.05.

#### 2.7.2 NMA

STATA16.0 software will be used to through the GeMTC package will be used to perform NMA to synthesize direct and indirect evidence. The NMA will be undertaken primarily in WinBUGS1.4.3 software, using the Bayesian Markov Chain Monte Carlo random effect model.^[22]^ The convergence of the stimulation is tested by the potential scale reduction parameter, when the closer potential scale reduction parameter will be to 1, the better the model convergence is and more reliable the conclusion is.^[23]^ The selection of the final model will depend on the deviance information criterion value. Generally, a model with a smaller deviance information criterion value is better.^[24]^ Numerical variables will be presented as standardized mean differences with 95% CI. We will draw a surface under the cumulative ranking (SUCRA) for each outcome indicator to predict the order of curative effect of treatment measures, the larger the area under the curve, the better the treatment measures.

We will conduct the consistency model analysis of the primary outcome indexes and the probability ranking of the best treatment measures. If there is a closed ring, the node splitting method will be used to evaluate the inconsistency of each loop.^[25,26]^

#### 2.7.3 Sensitivity analysis and subgroup analysis

We will explore sources of heterogeneity by performing a network meta-regression using a random-effects network meta-regression model. If the necessary data are available, subgroup analysis will be carried out according to different factors as follows: the duration or dosage of ACU, duration of disease, period of treatment, and the type of intervention in the control group. A sensitivity analysis will be performed when there is significant heterogeneity according to the following aspects: sample size, heterogeneity qualities, methodological elements, and characteristic of research. If heterogeneity is reduced after low-quality or small sample studies are excluded, and we must be more cautious in concluding.

#### 2.7.4 Publication bias

Publication bias will be evaluated using an Egger regression test which will help avoid observation bias and produce a funnel plot indicating a digitally based modeling result.

#### 2.7.5 Grading the quality of evidence

The grading of evidence quality will be conducted by 2 independent reviewers using the grading of recommendations assessment, development, and evaluation instrument.^[27]^ All studies will be rated as 4 levels as following: high, moderate, low or very low, which according to the 5 aspects (inconsistency, limitations, imprecision, indirectness, and publication bias).^[28]^

## 3 Discussion

ACU as an effective technique of TCM has been accepted for MGH in China. Still, due to the lack of direct comparison between different ACU methods, clinicians cannot choose the best one. As a result, clinicians tend to combine several ACU-based methods from their experience to determine which are most suitable for patients, which increases the burden of time and capital input, and results in inefficient utilization of medical resources. NMA can be used to integrate direct and indirect comparisons,^[29]^ which can make clear about the inferences of efficacy and help to compare efficacy of available therapies.^[30]^ Therefore, we would like to see an NMA, which is made in strict accordance with the NMA procedures, so as to provide more evidence to support the beneficial use of ACU and encourage more extensive use of ACU as an alternative therapy for MGH.

The study also has some defects as follows: low quality of original researches, possible occurrence of false positive or false negative results, various duration of disease, different dosage and frequency of intervention, language restriction, and so on. All of these will lead to some bias and influence the results of evaluation, which may ultimately affect the reliability of this study.

## Author contributions

**Conceptualization:** Fanghui Hua, Jun Xiong.

**Data curation:** Fanghui Hua, Jie Xiang, Yunfeng Jiang, Shouqiang Huang, Kai Liao.

**Formal analysis:** Shouqiang Huang, Jie Xiang.

**Investigation:** Jun Xiong, Honglian Li.

**Methodology:** XiaoHong Zhou, Shouqiang Huang, Jie Xiang, Meihua Liu.

**Software:** Kai Liao, Honglian Li, Meihua Liu.

**Supervision:** Jun Xiong, Honglian Li, Yunfeng Jiang.

**Writing – original draft:** Jun Xiong, Honglian Li.

**Writing – review & editing:** Jun Xiong, Honglian Li.
